# Zn-dependent β-amyloid Aggregation and its Reversal by the Tetrapeptide HAEE

**DOI:** 10.14336/AD.2022.0827

**Published:** 2023-04-01

**Authors:** Vladimir A Mitkevich, Evgeny P Barykin, Svetlana Eremina, Bibhusita Pani, Olga Katkova-Zhukotskaya, Vladimir I Polshakov, Alexei A Adzhubei, Sergey A Kozin, Alexander S Mironov, Alexander A Makarov, Evgeny Nudler

**Affiliations:** ^1^Engelhardt Institute of Molecular Biology, Russian Academy of Sciences, Moscow, Russia.; ^2^Department of Biochemistry and Molecular Pharmacology, New York University Grossman School of Medicine, New York, USA.; ^3^Faculty of Fundamental Medicine, M.V. Lomonosov Moscow State University, Moscow, Russia.; ^4^Washington University School of Medicine and Health Sciences, Washington, DC, USA.; ^5^Howard Hughes Medical Institute, New York University Grossman School of Medicine, New York, USA.

**Keywords:** Alzheimer disease, Aβ-Peptides;, Isoaspartatic acid, Zinc, *Caenorhabditis* elegans

## Abstract

The pathogenesis of Alzheimer's disease (AD) is associated with the formation of cerebral amyloid plaques, the main components of which are the modified Aβ molecules as well as the metal ions. Aβ isomerized at Asp7 residue (isoD7-Aβ) is the most abundant isoform in amyloid plaques. We hypothesized that the pathogenic effect of isoD7-Aβ is due to the formation of zinc-dependent oligomers, and that this interaction can be disrupted by the rationally designed tetrapeptide (HAEE). Here, we utilized surface plasmon resonance, nuclear magnetic resonance, and molecular dynamics simulation to demonstrate Zn^2+^-dependent oligomerization of isoD7-Aβ and the formation of a stable isoD7-Aβ:Zn^2+^:HAEE complex incapable of forming oligomers. To demonstrate the physiological importance of zinc-dependent isoD7-Aβ oligomerization and the ability of HAEE to interfere with this process at the organismal level, we employed transgenic nematodes overexpressing human Aβ. We show that the presence of isoD7-Aβ in the medium triggers extensive amyloidosis that occurs in a Zn^2+^-dependent manner, enhances paralysis, and shortens the animals’ lifespan. Exogenous HAEE completely reverses these pathological effects of isoD7-Aβ. We conclude that the synergistic action of isoD7-Aβ and Zn^2+^ promotes Aβ aggregation and that the selected small molecules capable of interrupting this process, such as HAEE, can potentially serve as anti-amyloid therapeutics.

The development of AD comprises a cascade of pathogenic processes in brain tissue. Some of the major elements of this cascade are the aggregation of beta-amyloid (Aβ) with the formation of neurotoxic oligomers, hyperphosphorylation of tau, and neuroinflammation [1]. As a result, the characteristic histopathological picture of AD exhibits extracellular amyloid plaques and intracellular tau tangles. In the sporadic form of AD, accounting for 95% of disease cases, the molecular triggers for the pathological cascade remain unknown. Evidently, these triggers are age-related and affect the Aβ metabolism. Post-translationally modified forms of Aβ, the most common of which, is isomerized Asp7 (isoD7-Aβ) [2-4], are the candidates for such triggers. Isomerization of Asp is a spontaneous modification that accumulates over time during proteostasis decline [2, 5, 6]. In contrast to intact Aβ, intravenously administered isoD7-Aβ sharply accelerates cerebral amyloidosis in transgenic mice overexpressing human Aβ, potentially acting as an amyloid seeding. Moreover, synthetic isoD7-Aβ, rather than intact Aβ, causes a significantly higher level of tau phosphorylation in cell culture [7]. These findings suggest the role of isoD7-Aβ as a molecular trigger for the pathogenic cascade of AD and a potential drug target. Indeed, it has been recently shown that targeting isoD7-Aβ with an antibody attenuates AD-like pathology in transgenic mice [8]. The pathological properties of isoD7-Aβ may be a result of its enhanced ability to form oligomers in the presence of zinc ions [9, 10]. Zn^2+^ promotes the accumulation of Aβ oligomers in synapses in a neuronal activity-dependent manner, and of all the divalent cations, it plays the most significant role in the formation of senile plaques [11, 12]. However, there is no experimental data implicating the interaction of Zn^2+^ and isoD7-Aβ in AD pathology.

In this study, we utilized the *C. elegans* model of Aβ amyloidosis to study the effect of isoD7-Aβ and zinc ions on animal pathophysiology and aging. We show that the concurrent administration of Zn^2+^ and isoD7-Aβ leads to a significant increase in amyloidosis accompanied by the shortening of animals’ lifespan. We further demonstrate that the tetrapeptide, HAEE, previously designed to counter the receptor toxicity of Aβ *in vitro* [13], can prevent zinc-induced oligomerization of isoD7-Aβ, negate the pro-amyloid effects of isoD7-Aβ:Zn^2+^ in live animals, and restore their lifespan. Our surface plasmon resonance (SPR), nuclear magnetic resonance (NMR), and molecular dynamics (MD) studies indicate that the molecular mechanism underlying the anti-amyloid effect of HAEE relies on its specific zinc-dependent and stable binding to Aβ and isoD7-Aβ. Together, these results elucidate the fundamental role of non-covalent complexes between the zinc ion and isoD7-Aβ in triggering the pathological aggregation of endogenous Aβ molecules and suggest that the compounds targeting such complexes have a therapeutic potential.

## MATERIALS AND METHODS

### Materials

All chemicals and solvents were of HPLC-grade or better and were obtained from Sigma-Aldrich (St. Louis, MO, USA). Synthetic peptides (purity > 98% checked by RP-HPLC) Aβ_16_ (Ac-DAEFRHDSGYEVHHQK-NH_2_), H6R -Aβ_1-16_ (Ac-DAEFRRDSGYEVHHQK-NH_2_), isoD7-Aβ_16_ (Ac-DAEFRH[isoD]SGYEVHHQK-NH_2_, where [isoD] - isoaspartate), Aβ_42_ (DAEFRHDSGYEVHHQKL VFFAEDVGSNKGAIIGLMVGGVVIA), isoD7-Aβ_42_ (DAEFRH[isoD]SGYEVHHQKLVFFAEDVGSNKGAIIGLMVGGVVIA), GGGGC-Aβ_42_, GGGGC-isoD7-Aβ_42_, and Ac-HAEE-NH_2_ were purchased from Biopeptide Co., LLC (San Diego, CA, USA). Zinc chloride (99.99%, ACROS Organics) was dried out for 1-2 hours at 150°C prior to weighing.

### SPR analysis

**Figure 1.**
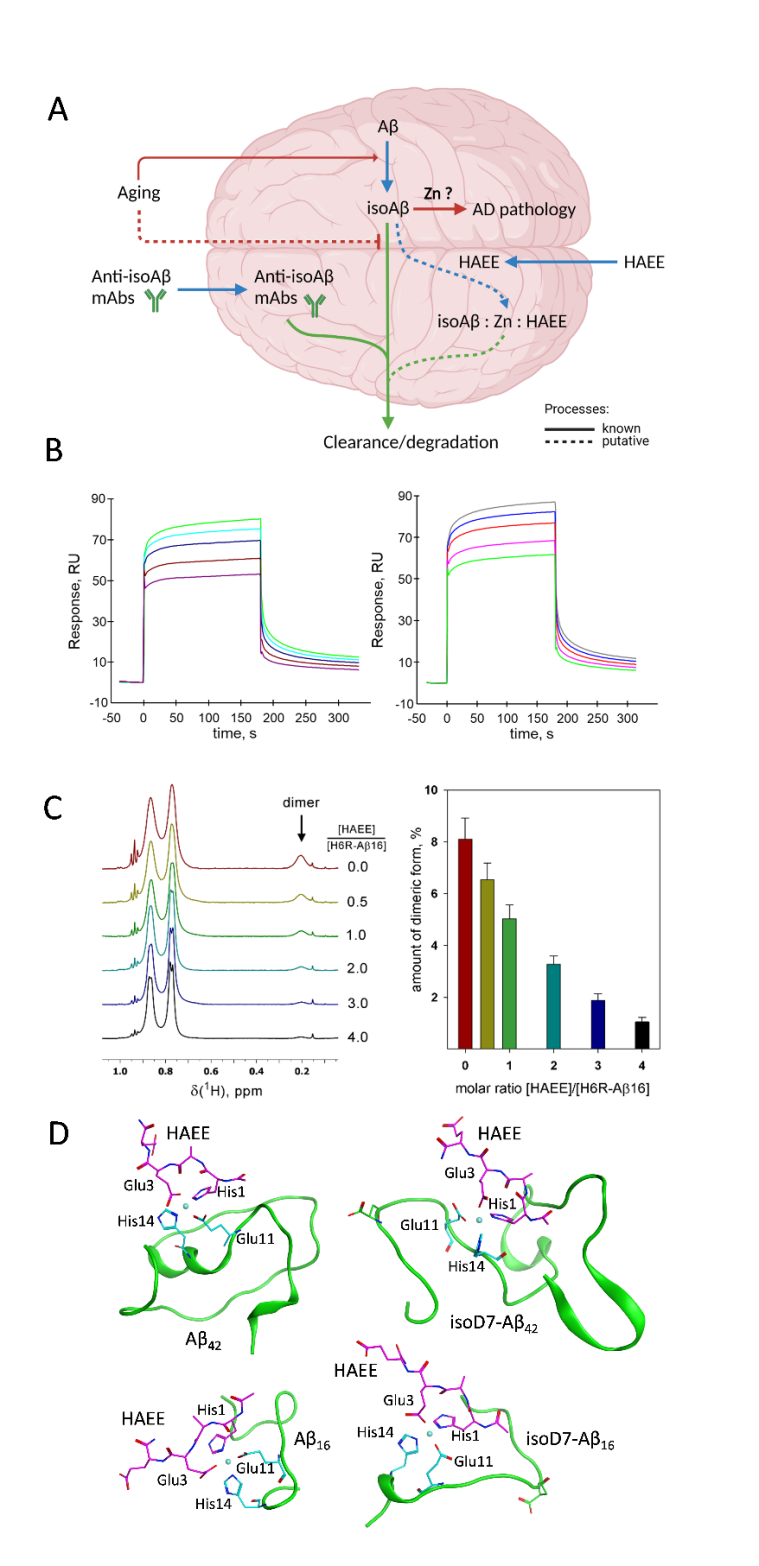
Interaction of HAEE with beta-amyloid. (A) A model for the contribution of isoD7-Aβ to AD pathology, and the possible ways of preventing its deleterious effects. Aβ spontaneously converts into isoD7-Aβ, which is accumulated over age, likely due to reduced Aβ clearance. Isomerized Aβ promotes AD pathology, potentially via formation of Zn-bound complexes serving as the seeds for a generation of neurotoxic oligomers. Treatment with anti-isoAβ antibodies is a promising therapeutic strategy already showing effectiveness in AD model mice ^9^. HAEE was shown to hamper amyloid accumulation in animal models of AD ^17^, and its effect may be due to the formation of harmless isoAβ:Zn^2+^:HAEE complexes, which are subject to degradation. (B) A set of sensorgrams illustrating paired interactions of various concentrations of HAEE (150, 300, 500, 1000, 1500 μM) with immobilized Aβ_42_ (left panel) and isoD7-Aβ_42_ (right panel) in the presence of 100 µM ZnCl_2_, pH 6.8. (C) The addition of HAEE leads to a decrease in the amount of dimeric H6R-Aβ_16_. Left panel: fragments of ^1^H NMR spectra of a solution of the H6R-Aβ_16_ peptide at a concentration of 1.6 mM, pH 6.8 in the presence of the 0.5 equivalent of ZnCl_2_ during titration by HAEE. Right panel: a decrease in the content of dimeric H6R-Aβ_16_ upon addition of HAEE. The amount of dimeric form is determined by the intensity of its characteristic signal at 0.2 ppm. (D) Interaction interface between HAEE (magenta) and Aβ_42_, Aβ_16_ (green) via zinc (left) and between HAEE (magenta) and isoD7-Aβ_42_, isoD7-Aβ_16_ (green) via zinc (right). Representative structures after 100-200 ns of MD simulation. Zinc is coordinated by oxygen atoms of glutamic acid and nitrogen atoms of histidine rings. ^11^EVHH^14^ atoms are colored in cyan.

All SPR measurements were carried out at 25°C using an optical biosensor Biacore 8K (GE Healthcare, USA) with CM4 optical chip. Covalent immobilization of the peptide ligands on the surface of the optical chip was performed using the sulfhydryl group of the C-terminal tetragly-cylcysteine tag. Briefly, carboxyl groups on the chip surface were activated by serial injections of mixture of 0.4 M EDC/0.1 M NHS at a flow rate of 10 μL/min for 2 min and a solution of 80 mM PDEA in 100 mM borate buffer (pH 8.5) for 4 min. Solutions of 2 μM peptides in the immobilization buffer (10 mM acetate buffer, pH 4.5) were injected for 2 min at a flow rate of 10 μL/min (three repeats). Unreacted activated groups on the chip were blocked by further injection of a solution containing 50 mM cysteine, 1 M NaCl, 0.1 M sodium acetate (pH 4.3) for 4 min at a flow rate of 5 μL/min. Possible nonspecific analyte binding to the chip surface was evaluated using a free (control) channel of the biosensor exposed to the same treatments as the working channel except peptides. SPR signals were recorded in real time in resonance units (RU; 1 RU corresponds to 1 pg of analyte) and were presented in the form of sensorgrams showing time-dependent signal changes. A series of sensorgrams representing the difference of SPR-signals from working and control channels were obtained by serial injections of analyte solutions (Fig. 1B) through the working and control channels at a flow rate of 30 μL/min for 3 min. All SPR measurements were repeated 3 times. After each measurement the optical chip surface was regenerated by injecting an HBS-EP (0.01 M HEPES pH 7.4, 0.15 M NaCl, 3 mM EDTA, 0.005% v/v Surfactant P20) buffer for 30 s. Analyte samples were prepared in the running buffer (10 mM HEPES, pH 6.8 in the absence of Zn^2+^ and 10 mM HEPES, 100 µM ZnCl_2_, pH 6.8). The obtained sensorgrams were analyzed with BIAevaluation v.4.1 software.

### NMR experiments

Peptides Aβ_16_, H6R-Aβ_16_ and isoD7-Aβ_16_ at the concentration of 0.2 - 8mM were dissolved in 10-20 mM bis-Tris-d_19_ (2,2-Bis(hydroxymethyl)-2,2′,2"-nitrilotrie-thanol-d_19_ with 98% ^2^D enrichment) buffer solution (pH 6.8). Sodium salt of 3-(trimethylsilyl) propionic-2,2,3,3-d_4_ acid (DSS) at a concentration of 10-20 μM was added as the standard. NMR spectra were measured at 278 K in 90% H_2_O/10% D_2_O with Bruker AVANCE 600 MHz spectrometer equipped with triple resonance (^1^H, ^13^C and ^15^N) pulsed field z gradient probe. 1D NMR spectra were processed and analyzed using the Mnova software (Mestrelab Research, Spain). In order to monitor peptide-peptide interactions NMR titration experiments were carried out. Peptides at a concentration of 0.2-2.0 mM at pH 6.8 were titrated with a solution of HAEE and ZnCl_2_ in a buffer of identical composition at the same pH value. 1D spectra were recorded for each titration point.

### Modeling and MD simulations of the isoD7-Aβ:HAEE interactions

The templates for amyloid beta-peptide isoD7-Aβ_16_ and HAEE structure modeling with zinc were taken from the PDB:2MGT solution NMR structure of zinc-induced dimer of the human amyloid beta-peptide metal binding domain 1-16 with Alzheimer's disease pathogenic English mutation H6R [9]. To obtain the isoD7-Aβ_16_ structure, in one of the H6R-Aβ_1-16_ molecules in the PDB:2MGT dimer, Asp7 was replaced with iso-asparagine and Arg6 was replaced with histidine, thus, Aβ_16_ reverted to the iso-Asp7 primary structure. In the initial HAEE structure, hydrogens, acetyl, and amino (CH_3_CO and NH_2_ respectively) end groups were added and the resulting structure was minimized in water and relaxed for 100 ns using the Gromacs package [14]. HAEE was superposed with the structure of the second H6R-Aβ_16_ molecule in the PDB:2MGT dimer, so that the positions of arginine and histidine atoms of Aβ_16_ and HAEE, which coordinate zinc, corresponded as much as possible. This initial structure, after isoD7-Aβ_16_ was replaced by isoD7-Aβ_42_, was also used to model the isoD7-Aβ_42_:HAEE interaction. The structure of isoD7-Aβ_42_ was modeled by us previously [15]. Expert modeling was used to align the position of His13 side chain in isoD7-Aβ_42_ with the corresponding side chain in the template zinc coordination interface. The resulting structures were solvated with TIP3P water and NaCl ions at a concentration of 115 mM. To model a zinc ion, two different special force fields based on quantum calculations were used, with practically no difference in the resulting Aβ:HAEE structures [16, 17]. The parametrization of the Aβ, HAEE, Glu, and His residues in coordinating zinc have been changed in accordance with the above force field. After 200 ps equilibration in NVT and NPT ensembles respectively the systems were simulated for 100 ns of MD production run at 300 K. All simulations were carried out using the particle mesh Ewald technique with repeating boundary conditions and 1 nm cut-offs, using the LINCS constraint algorithm with a 2 fs time step. The final structure after completion of MD simulation was taken as the representative structure. To analyze the stability of the Aβ final conformations, root mean square distance (RMSD) and root mean square fluctuation (RMSF) calculations of the MD trajectories were performed. The RMSD plot shows conformational stability of the structure during the MD simulation relevant to the reference structure.

### Strains and growth conditions

*C. elegans* CL2120 (dvIs14 [(pCL12) unc-54::beta 1-42 +(pCL26) mtl-2::GFP]) and *C. elegans* CL2122 (dvIs15 [(pPD30.38) unc-54(vector) + (pCL26) mtl-2::GFP]) strains were obtained from the Caenorhabditis Genetics Center. The worms were handled according to standard methods without FUDR [18]. *E. coli* OP50 bacteria were grown overnight in Luria-Bertani broth and 50µl were spread atop NGM or NGMZn plates. NGMZn has 20μM ZnSO_4_. Seeded plates were incubated at 30°C for ~16 hours and then for 1-2 hours at 15 or 25°C before worms were transferred. Lifespan and paralysis experiments (see below) were performed at 25°C for enhanced expression of Aβ. This temperature is standard when studying the effects of Aβ on *C. elegans* CL2120 strain [19].

### Treatment with isoD7-Aβ_42_ peptide and HAEE

Nematodes were purified from unwanted microflora by the alkaline hypochlorite method, grown to the L4 stage at 15°C on NGM plates with bacterial lawn. Approximately 140 animals were then placed in a drop of 0.2 ml M9-buffer +/- ZnSO_4_ (20μM) +/- isoD7-Aβ_42_ (40μM) +/- HAEE (4mM) and cultured for 4 hours at 25°C by stirring. Further, all contents were transferred to Petri plates with NGM or NGMZn with OP50 and placed in a temperature-controlled incubator at 25°C. Next day, worms were transferred onto similar media with a bacterial substrate and the lifespan was determined at 25°C (Supplementary Tables 3, 4).

### Imaging and quantitative analysis of amyloid deposits

**Figure 2.**
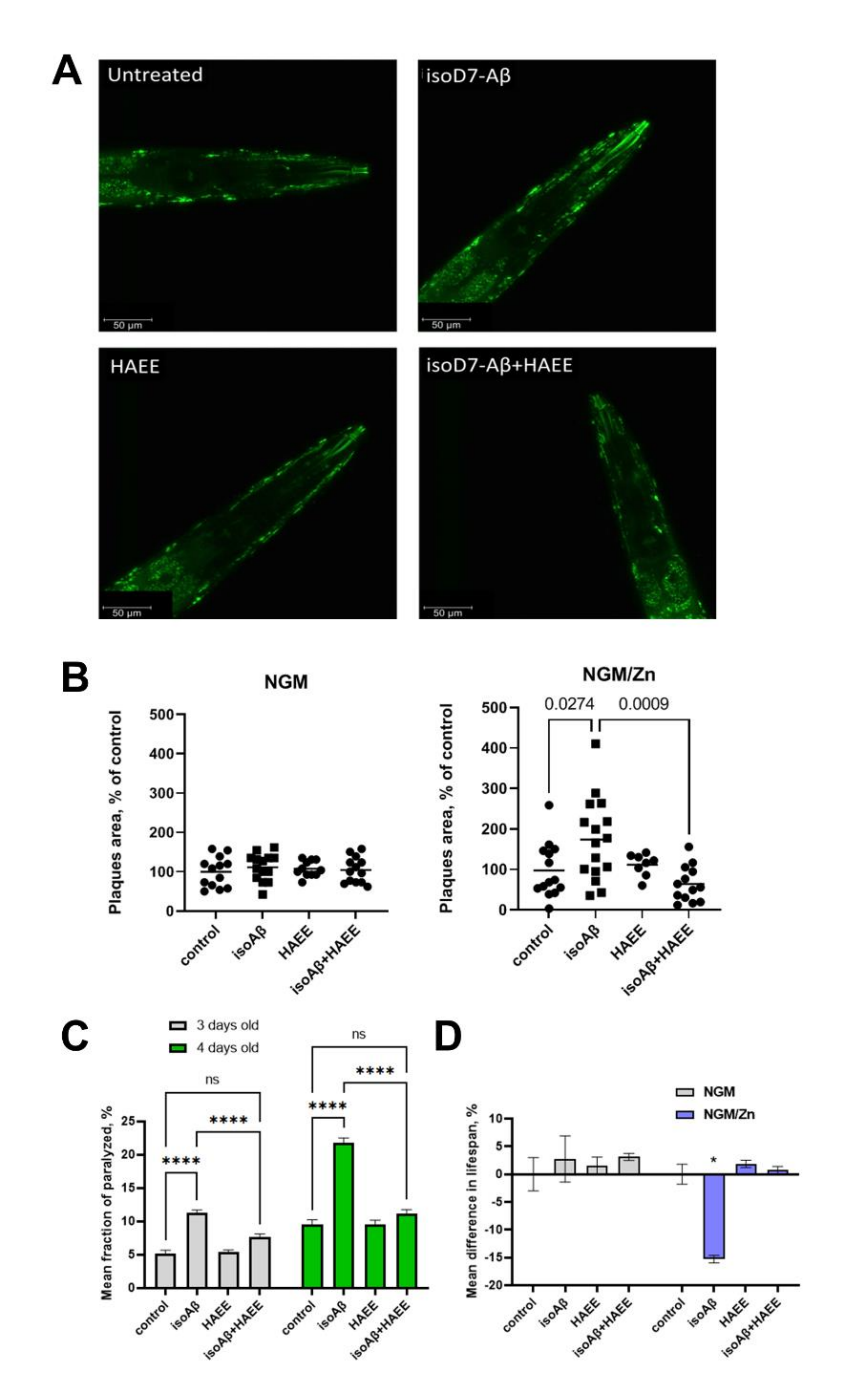
The effect of isoD7-Aβ_42_ and HAEE in the presence of Zn^2+^ on amyloid load in *C. elegans*. (A) A visualization of amyloid aggregates in a *C. elegans* CL2120 strain untreated or incubated with isoD7-Aβ_42_, HAEE, or isoD7-Aβ_42_ and HAEE combined. Amyloid aggregates were stained with fluorescent dye X-34 in live animals. Shown are the maximum projections of z-stack images acquired with a confocal microscope. (B) The area covered with amyloid aggregates (“plaques”) as a percentage of head area, detected by X-34 fluorescence. Worms were treated with 40 µM of isoD7-Aβ_42_ (isoAβ), 4 mM of HAEE, these two peptides combined (isoAβ+HAEE) or received no treatment (control). After the incubation with peptides on NGMZn, the animals were stained with X-34 and amyloid aggregates were visualized with a confocal microscope. Data shown as individual values with a bar at sample mean. N for control, isoAβ, HAEE, isoAβ+HAEE treated worms equals 13, 15, 11, 13 for NGM group and 14, 16, 8, 12 for NGM/Zn group, respectively. Brackets represent statistically significant comparisons according to ANOVA with post-hoc Tukey test. P-values are indicated above the brackets. (C) The effects of isoD7-Aβ_42_ and HAEE on the prevalence of a paralysis phenotype in *C. elegans* CL2120 of 3 days old (grey) and 4 days old (green). Treatment: 0.2 ml M9 buffer + ZnSO_4_ (20μM) +/- isoD7-Aβ_42_ (40 μM) +/- HAEE (4 mM). Strains were grown on NGMZn. Data is shown as the mean of 3 independent experiments ± SD. Brackets represent statistically significant comparisons according to ANOVA with post-hoc Tukey test. **** - p<0.0001, ns - non-significant. (D) The effects of isoD7-Aβ_42_ and HAEE on *C. elegans* CL2120 lifespan. Left graph: Strains were grown on NGM. Treatment: 0.2 ml M9 buffer +/- isoD7-Aβ_42_ (40 μM) +/- HAEE (4 mM). Right graph: Strains were grown on NGMZn. Treatment: 0.2 ml M9 buffer + ZnSO_4_ (20μM) +/- isoD7-Aβ_42_ (40μM) +/- HAEE (4 mM). Data is shown as the mean difference in lifespan ± SD (%) of treated worms compared to control. The results of 3 independent experiments are presented. * - p<0.0001, according to Student t test.

Nematodes were grown on NGM or NGMZn medium with a *E. coli* OP50 strain at 25°C and treated with amyloid peptides and/or HAEE as described above. At the A1 stage, the animals were stained with 1 mM X-34 (Sigma-Aldrich, St. Louis, MO, USA) in 10 mM Tris pH 8.0 for two hours. For de-staining, they were afterwards cultured for 16 hours on NGM or NGMZn with a bacterial lawn at the same temperature. Stained individual worms were mounted on glass slides in agar and anesthetized by incubation in 0.1% Na_3_N. Amyloid aggregates were visualized with Leica TCS SP5 confocal microscope (Leica Microsystems GmbH, Wetzlar, Germany) using 405 nm laser for excitation and a 430-570 bandpass emission filter with 63x oil immersion objective. Worms were imaged as z-stacks of 10-15 images 4.1 µm thickness each. For imaging, standard Las X software (Leica Microsystems GmbH, Wetzlar, Germany) with a plugin for confocal imaging was used. Acquired z-stacks were processed with Las X software to obtain maximum projections. Background subtraction and thresholding were performed by WCIF ImageJ with identical parameters for all images. Total plaque area, mean fluorescence intensity and plaques count were quantified for each worm in an area spanning from anterior end to posterior bulb. Data for plaque area is presented on graphs as individual values normalized by the amyloid load in control with bars representing sample means. The comparison of data groups was performed with ordinary one-way ANOVA. Post-hoc analysis was performed with the Tukey test. The Shapiro-Wilk test was used to confirm the normality of the dataset. Statistical analysis was performed with GraphPad Prism 9.1 software (GraphPad Software Inc., CA, USA). The representative nematode images for Fig. 2A were selected to have the plaques area that is close to the mean of each sample.

### Paralysis assay

Synchronized eggs of CL2120 were grown to the L4 stage at 15°C on NGM plates with bacterial lawn, were treated with ZnSO_4_, Aβ_42_ (40 μM), isoD7-Aβ_42_ (40μM), HAEE (4mM) and isoD7-Aβ_42_ with HAEE at 25°C. Then, nematodes were transferred to Petri plates with NGM or NGMZn and OP-50, placed in a temperature-controlled incubator at 25°C. On the next day, worms were transferred onto similar media with a bacterial substrate and paralyzed nematodes were counted at equal time intervals. The worms that only moved their head or did not show a full-body movement when gently touched with a platinum loop were scored as paralyzed. For the paralysis experiments, we chose the days where we could clearly distinguish between paralysis and mortality. This conformed to the third and fourth days of their adult life. While at least three repeated trials were conducted, each experiment was performed using at least 100 worms. Details regarding repeated experiments and amounts of animals used for experiments are summarized in Supplementary Table 2. Mean fraction of paralyzed worms was compared between groups with two-way ANOVA, accounting for age and treatment. Post-hoc analysis was performed with the Tukey test. Statistical analysis was performed with GraphPad Prism 9.1 software.

### Lifespan analysis

Lifespans were monitored at 25°C as described previously [20, 21]. All experiments were repeated at least three times and ≈130 worms were used for each experiment. Details regarding repeated experiments and amounts of animals used for experiments are summarized in Supplementary Tables 3, 4. In all cases, stage L4 worms were used at t = 0 for lifespan analyses and worms were transferred every other day to new agar plates. Worms were judged as dead when they ceased pharyngeal pumping and did not respond to prodding with a platinum wire. Escaped animals or animals with internal hatching were not included in lifespan calculation. Data was analyzed and Boltzmann sigmoid survival curves generated using the SciDAVis statistical analysis software package. Mean lifespans were compared in Microsoft Excel using the Student t test, applying one-tailed distribution and two-sample equal variance [22]. All lifespan plots represent the composites of all independent experiments tabulated in Supplementary Tables 3, 4. Mean percentage change ±SD of lifespan after treatment, relative to untreated control are indicated in each graph in the same color as the curve.

### Statistical analysis

The applied statistical tests and software for each type of data are specified in the respective Methods subsections. For all of the data, before applying parametric statistical tests, the D’Agostino & Pearson normality test in GraphPad Prism 9.1 software was used to demonstrate that all sample data is distributed normally.

## RESULTS

### HAEE stably interacts with Aβ and interferes with its Zn^2+^-dependent dimerization

In contrast to normal Aβ, both the full-length isomerized Aβ, isoD7-Aβ_42_, and its metal-binding fragment, isoD7-Aβ_16_, stimulate amyloidosis in transgenic mice [23, 24]. This correlates with the increased capacity of isoD7-Aβ_42_ and isoD7-Aβ_16_ for zinc-dependent aggregation [9]. We hypothesized that the contribution of isoD7-Aβ to the development of AD is based on the formation of zinc-bound complexes that promote pathological aggregation of Aβ (Fig. 1A). To test this hypothesis, we utilized a tool compound - the tetrapeptide HAEE, which was rationally designed to interact with the zinc-binding site of Aβ (^11^EVHH^14^) [13, 25].

We first used SPR analysis to study the interaction between the immobilized Aβ_42_ or isoD7-Aβ_42_ and the soluble HAEE tetrapeptide. In the absence of Zn^2+^, no interaction of HAEE with immobilized amyloid peptides was detected (Supplementary Fig. 1). In the presence of 100 µM ZnCl_2_, the sensorgrams of the HAEE interaction with both Aβ_42_ or isoD7-Aβ_42_ were obtained (Fig. 1B) and the interaction parameters calculated (Supplementary Table 1). The *K_D_* values for HAEE-Aβ_42_ and HAEE-isoD7-Aβ_42_ complexes were estimated to be 4.1±0.3 µM and 10.4±0.4 µM, respectively.

Next, we used NMR spectroscopy to obtain structural information on the interaction of HAEE with Aβ and isoD7-Aβ. Full-size Aβ quickly aggregates in solution in the presence of Zn^2+^ [26]. Therefore, we used a metal-binding fragment of Aβ (1-16 aa), which is an established model to study the role of Zn^2+^ in Aβ interactions. In the presence of Zn^2+^ the HAEE-induced chemical shift of Aβ_16_ (Supplementary Fig. 2) and isoD7-Aβ_16_ (Supplementary Fig. 3) signal was clearly observed. The presence of the tetrapeptide eliminated the signal at 0.2 ppm, which is characteristic of the dimeric form of the Aβ metal binding domain [9]. Thus, we conclude that HAEE inhibits the process of zinc-dependent dimerization in both Aβ_16_ and isoD7-Aβ_16_.

It should also be noted that the addition of Zn^2+^ to isoD7-Aβ_16_ leads to its almost complete oligomerization and precipitation [9], as evident in the disappearance of the corresponding NMR signal (Supplementary Fig. 3) However, in the presence of HAEE, Zn^2+^ did not diminish the isoD7-Aβ_16_ signal (Supplementary Fig. 3), highlighting the protective role of HAEE against its oligomerization.

It has been shown that an Aβ_1-16_ variant with the so-called English (H6R) mutation (H6R-Aβ_16_) exists as a mixture of monomeric and dimeric forms in the presence of Zn^2+^ [9]. The dimeric form has a well-resolved characteristic signal of the V12 methyl group at 0.2 ppm, which allows for a quantitative assessment of the dimeric state. The addition of HAEE to H6R-Aβ_16_ in the presence of Zn^2+^ leads to a disappearance of the dimeric form (Fig. 1C, Supplementary Fig. 4). Of note, the affinity of Zn^2+^ to HAEE is almost an order of magnitude less than for H6R-Aβ_16_ (Supplementary Fig. 5). Thus, the anti-dimerization effect of HAEE must be achieved via disrupting the H6R-Aβ_16_ dimerization interface, not by competing for zinc.

Next, we utilized molecular dynamics (MD) to model Zn^2+^-dependent interactions between HAEE and Aβ or its isoD7 isoform. MD simulations for 200 ns show the formation of stable complexes of Aβ_16_:HAEE, isoD7-Aβ_16_:HAEE, Aβ_42_:HAEE and isoD7-Aβ_42_:HAEE via the ^11^EVHH^14^ site coordinated by Zn^2+^ (Fig. 1D). In contrast, only transient interactions can be seen without Zn^2+^ (Supplementary Fig. 6). We performed MD modelling for a number of systems with protonated and neutral histidines in Aβ_1-16_ and HAEE and observed repeating multiple short-term interactions between HAEE and the ^11^EVHH^14^ site of Aβ_16_. In the isoD7-Aβ_16_:Zn^2+^:HAEE complex, the side chain interactions between Aβ Glu11/His14 and HAEE Glu3/His1 are coordinated by Zn^2+^ (Fig. 1D). In the case of isoD7-Aβ_42_, a similar complex was formed (Fig. 1D). Moreover, the C-terminus of isoD7-Aβ_42_ formed a loop, creating a cavity where Zn^2+^ interacted with HAEE. Additionally, four hydrogen bonds were formed between HAEE and His13, Asn27, Lys28 and Ala30 of isoD7-Aβ_42_. This structural feature contributed to the stability of the isoD7-Aβ_42_:Zn^2+^:HAEE complex.

To assess the stability of the isoD7-Aβ_16_:Zn^2+^:HAEE and isoD7-Aβ_42_:Zn^2+^:HAEE complexes, the RMSD values were calculated as a graph showing the divergence of a structure in every MD trajectory frame from the final structure (Supplementary Fig. 7). The final structures of complexes remained stable. As the isoD7-Aβ_42_:Zn^2+^:HAEE structure features 4 additional intramolecular hydrogen bonds, it less flexible than the isoD7-Aβ_16_:Zn^2+^:HAEE structure (Supplementary Fig. 8).

Our previous experimental studies [9] and MD modeling [27] showed that in zinc-induced aggregation of Aβ, the residues coordinating zinc in the ^11^EVHH^14^ site play a pivotal role. In this study, we show that the HAEE tetrapeptide efficiently binds these residues forming stable complexes, therefore these residues cannot participate in other interactions. Thus, we assume that the presence of HAEE tetrapeptide may prevent the aggregation of Aβ into unstructured conglomerates.

### Exogenous HAEE prevents isoD7-Aβ_42_:Zn^2+^-mediated accelerated amyloidosis in the C. elegans model of AD

To study the therapeutic potential of HAEE in mitigating isoD7-Aβ_42_:Zn^2+^-mediated pathology *in vivo*, we adopted an improved *C. elegans* model of Aβ_42_ toxicity (CL2120) that has been extensively used for AD research and drug screenings [28-32]. As the time required for a spontaneous isomerization of Asp7 in Aβ exceeds the lifespan of nematodes, it is highly unlikely that endogenous isoD7-Aβ would occur in CL2120 animals. Exogenous peptides, including Aβ-derived peptides, were previously shown to penetrate into *C. elegans* tissues upon addition to the growth or incubation medium [33-35], giving us an opportunity to study the effects of exogenous isoD7-Aβ and zinc ions on intrinsic Aβ_42_ pathology.

CL2120 and transgenic control (CL2122) nematodes were treated with Aβ_42_ or isoD7-Aβ_42_, with and without Zn^2+^, and amyloid aggregates visualized in live worms using amyloid-specific X-34 dye (Fig. 2A, Supplementary Fig. 9) [36]. In accordance with the previous data [28, 37], we observed age-dependent accumulation of Aβ_42_ in the muscle tissue of CL2120 nematodes, but not in the CL2122 control (Supplementary Fig. 9). In worms exposed to the isoD7-Aβ_42_/Zn^2+^ mixture, the area covered with plaques increased by 78% (Fig. 2A and B). Remarkably, the isoD7-Aβ_42_/Zn^2+^ - induced amyloidosis was completely negated in the presence of HAEE peptide, as both the number of plaques and plaque area reduced back to the untreated group values (Fig. 2A and B). Notably, animals treated with isoD7-Aβ_42_ in the absence of zinc, or with Aβ_42_, with or without Zn^2+^, did not show any increase in the amyloid load (Fig. 2B, Supplementary Fig. 10).

Thus, exogenous isoD7-Aβ_42_, not Aβ_42_, is capable of promoting Aβ_42_ amyloidosis in live animals in a Zn-dependent manner, whereas HAEE abrogates this pathological process.

### HAEE prevents isoD7-Aβ_42_:Zn^2+^-induced paralysis and decelerates aging in AD model animals

The deposition of amyloid aggregates reflects the increased concentration of toxic amyloid oligomers, which can be deleterious to surrounding tissue. In CL2120 nematodes Aβ_42_ is expressed in body wall muscle cells, resulting in severe age progressive-paralysis [28]. Therefore, we studied if the isoD7-Aβ_42_/Zn^2+^ mixture affects the paralysis of nematodes. We found that isoD7-Aβ_42_/Zn^2+^ accelerates paralysis in CL2120 animals, whereas HAEE suppresses this effect (Fig. 2C). The effect was not observed for Aβ_42_/Zn^2+^ nor amyloid peptides in the absence of Zn^2+^ (Supplementary Fig. 11, Supplementary Table 2).

Next, we studied how the lifespan of nematodes is affected by exogenous amyloid peptides and zinc. CL2120 and CL2122 animals have similar lifespans at 20°C [38]. The addition of Aβ_42_, with or without zinc ions, to NGM did not affect the mean lifespan of CL2120 or CL2122 animals (Supplementary Fig. 12, Supplementary Table 3). In contrast, the addition of the isoD7-Aβ_42_ peptide to Zn^2+^-containing media significantly decreased the lifespan of CL2120 animals, not control CL2122 animals (Fig. 2D, Supplementary Fig. 12, Supplementary Table 4). Without Zn^2+^, the negative effect of exogenous isoD7-Aβ_42_ on the Aβ_42_ transgenic animals was not detected.

Nematodes accumulate zinc in response to high zinc diet, and dietary zinc can be toxic to *C. elegans* [39, 40]. Taking into account the higher affinity of isoAsp7-containing peptide to zinc [10], it could promote toxicity by increasing the zinc uptake in a chelated form. However, the incubation medium contained only 20 µM of zinc, which is two orders of magnitude below the IC50 [39], thus arguing that the toxicity is due to isoD7-Aβ itself.

Strikingly, the addition of HAEE completely restored the shortened lifespan of isoD7-Aβ_42_ + Zn^2+^ - treated CL2120 animals. HAEE itself has no effect on the lifespan of CL2120 or CL2122 animals (Supplementary Fig. 13, Supplementary Table 4). Thus, we conclude that systemic amyloidosis promoted by exogenous isoD7-Aβ_42_ in the Zn-dependent manner shortens the animals’ lifespan, which can be cured by a rationally designed anti-isoD7-Aβ_42_ tetrapeptide (HAEE).

## DISCUSSION

Alzheimer's disease has a multifactorial pathology [41-45]. One of the main neuromorphological signs of AD is dense and diffused extracellular aggregates of Aβ - the amyloid plaques in brain tissue [1]. Accruing evidence suggests that the pathological cascade of AD is triggered by the accumulation of soluble neurotoxic Aβ oligomers [46]. In addition to the well-known effect of zinc ions on Aβ aggregation [11, 12], the formation of neurotoxic oligomers can be stimulated by the post-translational modifications of Aβ, such as Asp7 isomerization [8].

To identify the fundamental role of non-covalent complexes between Zn^2+^ and isoD7-Aβ in triggering pathological aggregation of endogenous Aβ molecules, we used the HAEE tetrapeptide as a specific molecular tool capable of binding Aβ and disrupting its metal-dependent dimerization interface. The formation of stable complexes of beta-amyloid peptides (^11^EVHH^14^ region) with HAEE, in the presence of Zn^2+^, is shown using SPR (Fig. 1B, Supplementary Table 1). Based on the structure of the zinc-bound H6R-Aβ_16_ dimer [9], models of the isoD7-Aβ complex with HAEE and Zn^2+^ for the full-length peptide and its metal-binding domain were created (Fig. 1D). Other potential Zn^2+^-interacting sites are predicted to further stabilize the complex via strong electrostatic forces, consistent with the results of the 200 ns MD run (Supplementary Fig. 7). NMR studies further show that HAEE shifts the equilibrium between monomeric and dimeric Aβ towards its monomeric form in the presence of zinc ions (Fig. 1C), indicating that it disrupts dimerization of Aβ via blocking the primary zinc-recognition site at ^11^EVHH^14^.

The *in vivo* data validates our mechanistic model of Zn^2+^-mediated amyloidosis. Strikingly, the amyloid burden in nematodes treated with isoD7-Aβ increased almost two-fold if zinc was present in the NGM medium (Fig. 2). The simultaneous presence of zinc ions and isoD7-Aβ in the diet also resulted in a significant enhancement in animal paralysis and shortening of the lifespan. If either zinc was absent from the medium or non-modified Aβ_42_ was used for the treatment, the changes in the amyloid load, paralysis, and lifespan were not detected.

It has been previously shown that, unlike Aβ_42_, exogenous isoD7-Aβ_42_ stimulates amyloidosis in transgenic mice, but the role of zinc in this process remained unknown. The present results imply that soluble Aβ species form zinc-bound complexes that enter *C. elegans* tissue. In the case of isoD7-Aβ_42_, such complexes promote amyloidosis and toxicity leading to animal paralysis and lifespan reduction. In the presence of HAEE, the combination of Zn^2+^ and isoD7-Aβ fails to induce amyloidosis and associated pathologies in *C. elegans* (Fig. 2), which demonstrates that oligomeric isoAβ:Zn assemblies trigger the morbific effect. Notably, the HAEE peptide did not affect the endogenous amyloidosis in untreated transgenic *C. elegans*.

Taken together, the experiments with *C. elegans* demonstrate, for the first time, the importance of both zinc ions and the isomerization of Asp7 for systemic toxic aggregation of native Aβ. They also identify HAEE as a small molecular agent capable of blocking this pathological process at the organismal level, suggesting a novel anti-amyloid therapeutic approach.

## Supplementary Materials

The Supplementary data can be found online at: www.aginganddisease.org/EN/10.14336/AD.2022.0827.

## Data and materials availability

The data that support the findings of this study are either included in the Manuscript and Supplementary information or available from the corresponding author upon reasonable request.
