# Supplementary material for: Zn-dependent β-amyloid Aggregation and its Reversal by the Tetrapeptide HAEE
Source: Aging Dis. 2023 Apr 1;14(2):309–18. doi: 10.14336/AD.2022.0827 (PMC10017155; doi:10.14336/AD.2022.0827)
Supplement: Supplementary file 1 — The Supplementary data can be found online at: www.aginganddisease.org/EN/10.14336/AD.2022.0827. [file AD-14-2-309-s.pdf]

SUPPLEMENTARY DATA

Zn-dependent β-amyloid Aggregation and its Reversal by the Tetrapeptide HAEE

Vladimir A. Mitkevich[1], Evgeny P. Barykin[1], Svetlana Eremina[1], Bibhusita Pani[2], Olga Katkova-Zhukotskaya[1], Vladimir I. Polshakov[3], Alexei A. Adzhubei[4], Sergey A. Kozin[1], Alexander S. Mironov[1], Alexander A. Makarov[1], Evgeny Nudler[2,5]*

© 2022. Mitkevich VA et al. Published online at http://www.aginganddisease.org/EN/10.14336/AD.2022.0827

SUPPLEMENTARY DATA

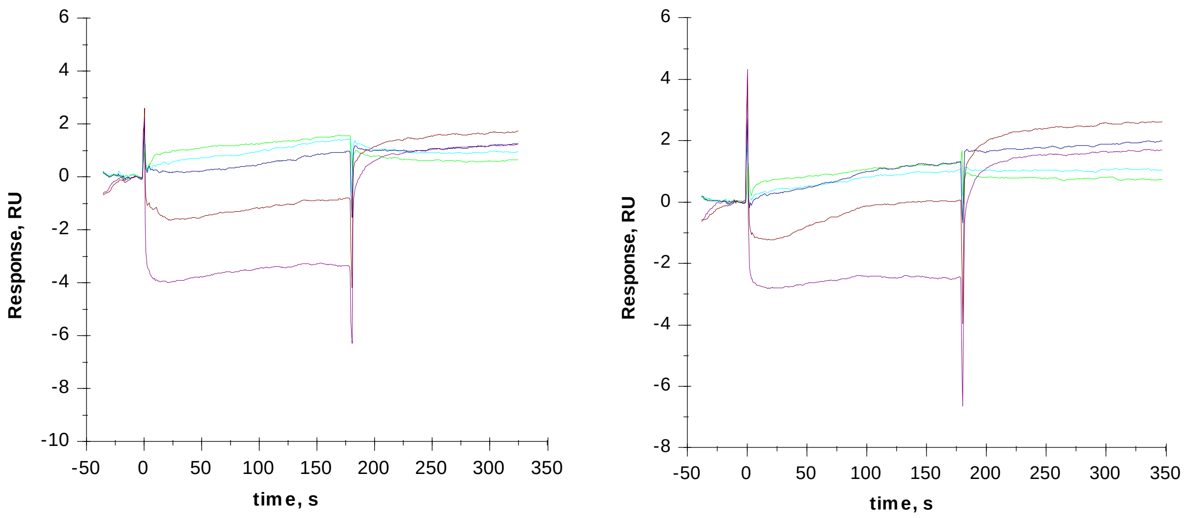

Supplementary Figure 1. A set of sensorgrams obtained by injection of different concentrations of HAEE (150, 300, 500, 1000, 1500 μM) to immobilized Aβ_{42} (left panel) and isoD7-Aβ_{42} (right panel) demonstrating the lack of interaction between peptides and HAEE in the absence of Zn^{2+}, pH 6.8.

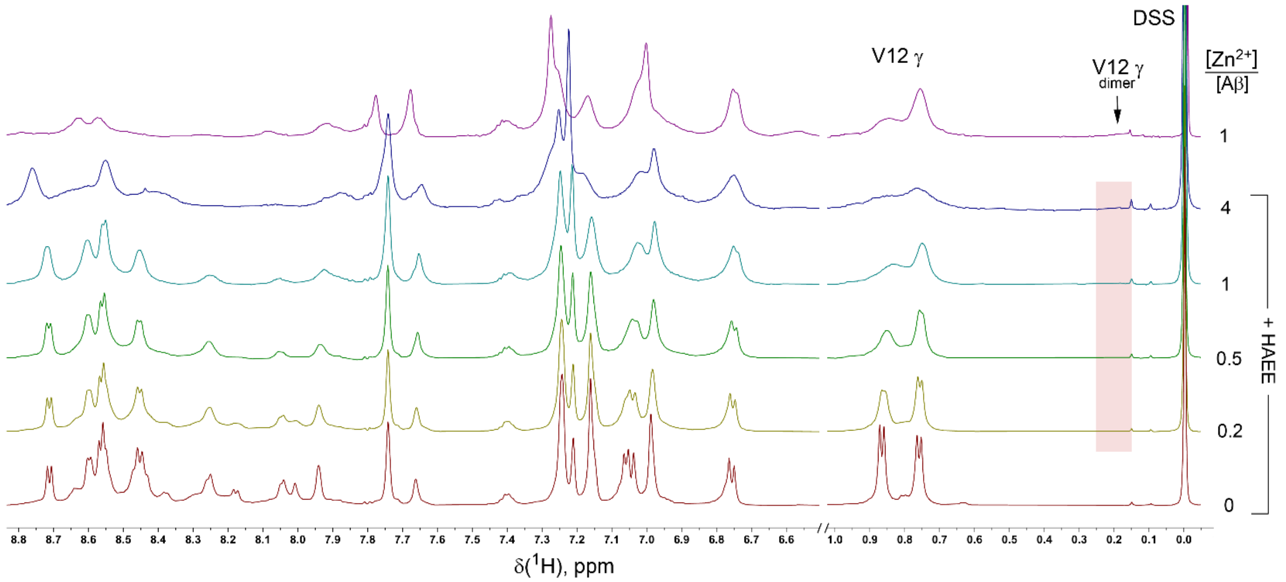

Supplementary Figure 2. Fragments of 1D NMR spectra of a mixture of Aβ_{16} and HAEE peptides in an equimolar ratio upon its titration with an increasing amount of zinc ions. (The molar ratio of Zn^{2+} ions to the peptides is shown to the right of the spectra.) Concentration of Aβ_{16} and HAEE is 0.3 mM. The regions of amide and aromatic signals, as well as the resonance region of the methyl groups of the V12 residue of the Aβ_{16} peptide are shown. The area of resonance of the signals of V12 methyl groups of the dimeric complex of the Aβ_{16} peptide with the zinc ion is highlighted in pink.

© 2022. Mitkevich VA et al. Published online at http://www.aginganddisease.org/EN/10.14336/AD.2022.0827

SUPPLEMENTARY DATA

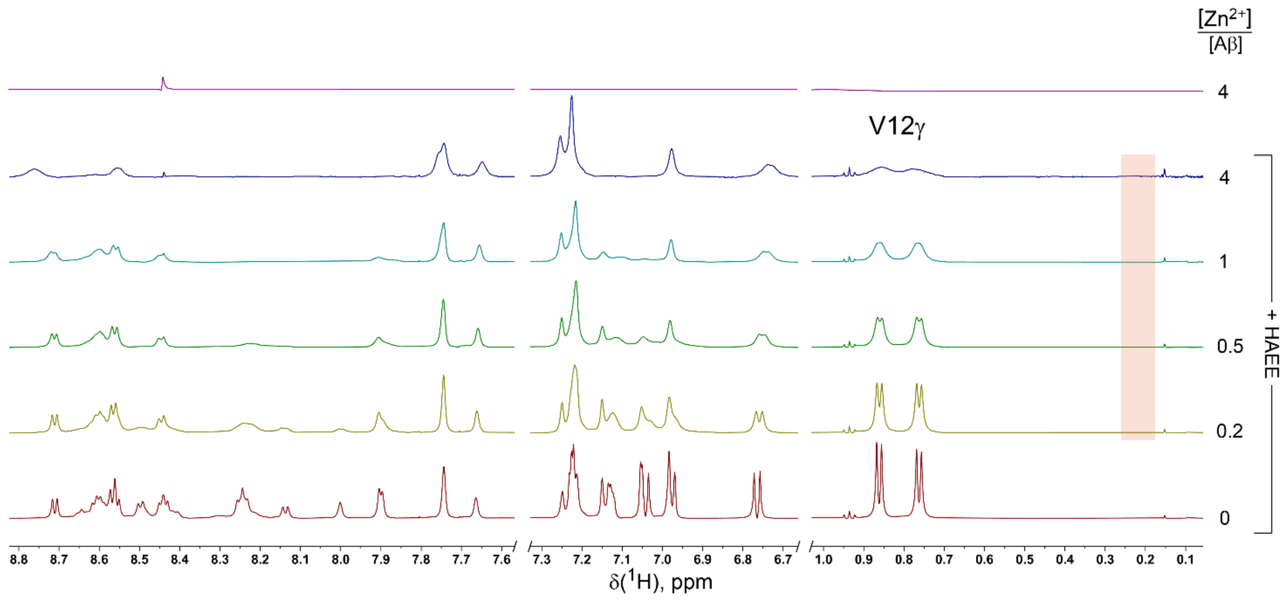

Supplementary Figure 3. Fragments of 1D NMR spectra of a mixture of isoD7-Aβ16 and HAEE peptides in an equimolar ratio upon its titration with an increasing amount of zinc ions. (The molar ratio of Zn^{2+} ions to the peptides is shown to the right of the spectra.) Concentration of isoD7-Aβ16 and HAEE is 0.3 mM. The regions of amide and aromatic signals, as well as the resonance region of the methyl groups of the V12 residue of the isoD7-Aβ16 peptide are shown. The area of resonance of the signals of V12 methyl groups of the dimeric complex of the isoD7-Aβ16 peptide with the zinc ion is highlighted in pink.

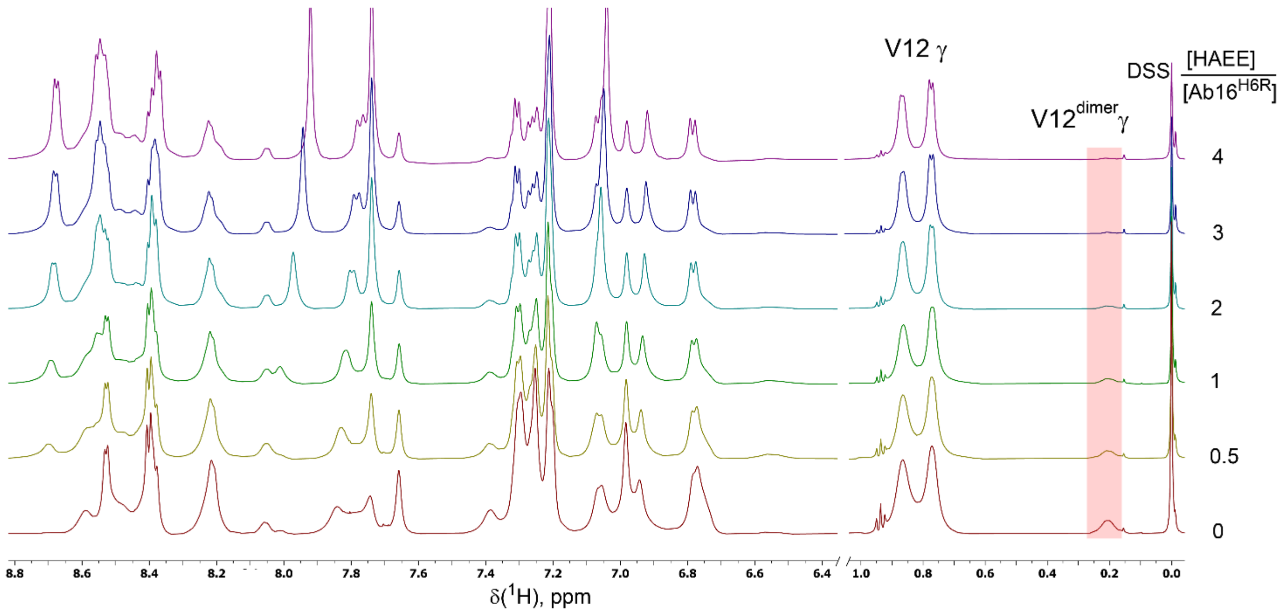

Supplementary Figure 4. Fragments of 1D NMR spectra of a solution of the H6R-Aβ16 peptide at a concentration of 1.6 mM, pH 6.8 in the presence of a 0.5 equivalent of $ZnCl_2$ during titration by HAEE. The molar ratio of HAEE to the H6R-Aβ16 peptide is shown to the right of the spectra. The regions of amide and aromatic signals, as well as the resonance region of the methyl groups of residue V12, are shown. The area of resonance of the signals of the V12 methyl groups of the dimeric complex of the H6R-Aβ16 peptide with the zinc ion is highlighted in pink.

© 2022. Mitkevich VA et al. Published online at http://www.aginganddisease.org/EN/10.14336/AD.2022.0827

SUPPLEMENTARY DATA

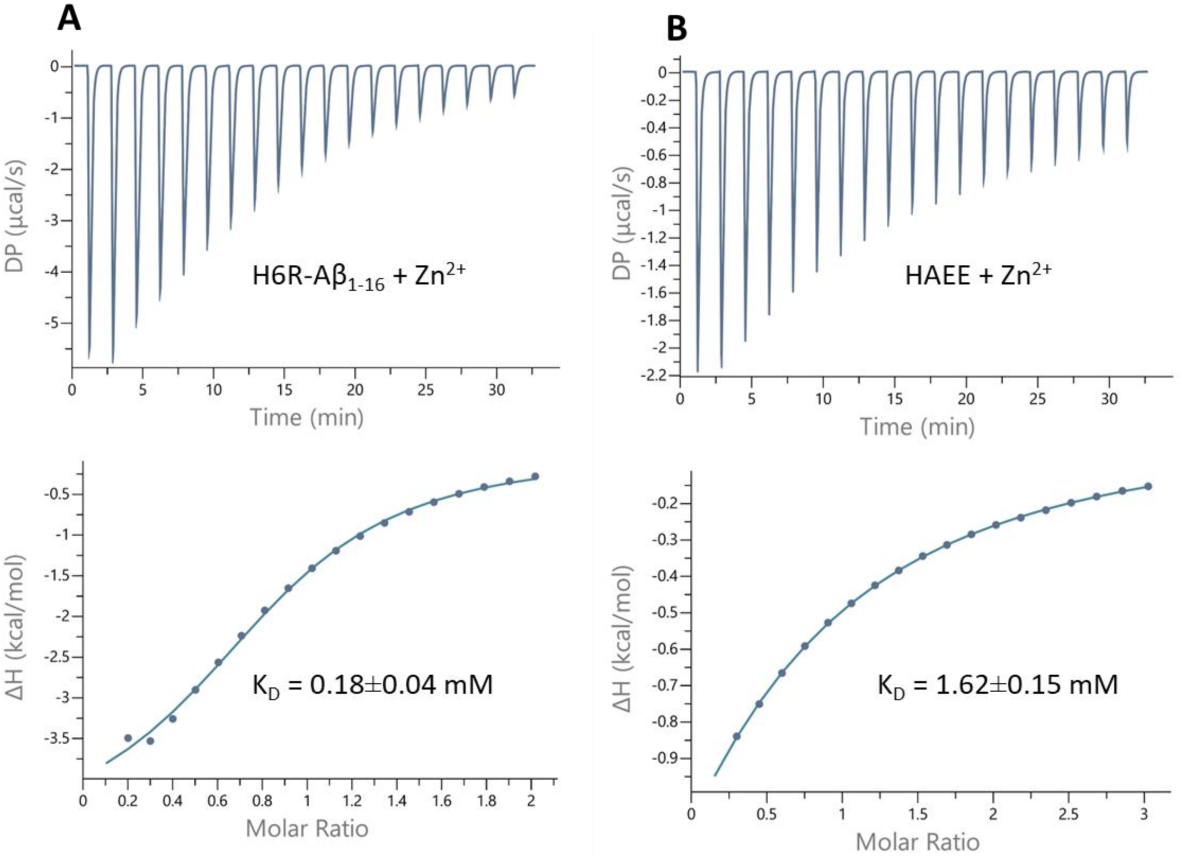

Supplementary Figure 5. Isothermal titration calorimetry titration curves (upper panels) and the binding isotherms (lower panels) for Zn^{2+} interactions with H6R-Aβ_{1-16} (**A**) and HAEE (**B**). Thermodynamic parameters of Zn^{2+} binding to H6R-Aβ_{1-16} and HAEE peptides were measured using a MicroCal PEAQ-ITC instrument (Malvern Panalytical, UK), as described previously [Tsvetkov P. O., Kulikova, A. A., Golovin, A. V., Tkachev, Y. V., Archakov, A. I., Kozin, S. A. & Makarov, A. A. (2010) Minimal Zn(2+) binding site of amyloid-beta. *Biophys. J.* 99:L84-6]. Experiments were carried out at 25°C in 50 mM HEPES buffer, pH 6.8. Aliquots of $ZnCl_2$ solution (2 l) were injected into the 0.2 ml cell containing solution of peptides to achieve a complete binding isotherm. Peptide concentration in the cell was 1-2 mM and the $ZnCl_2$ concentration in the syringe was 10-15 mM. Heat of dilution was measured by injecting the ligand ($ZnCl_2$) into the buffer solution; the obtained values were subtracted from the heat of reaction to calculate the effective heat of binding. The resulting titration curves were fitted using MicroCal PEAQ-ITC Analysis Software, assuming one set of binding sites. The dissociation constants (K_D) were determined by a non-linear regression fitting procedure.

© 2022. Mitkevich VA et al. Published online at http://www.aginganddisease.org/EN/10.14336/AD.2022.0827

SUPPLEMENTARY DATA

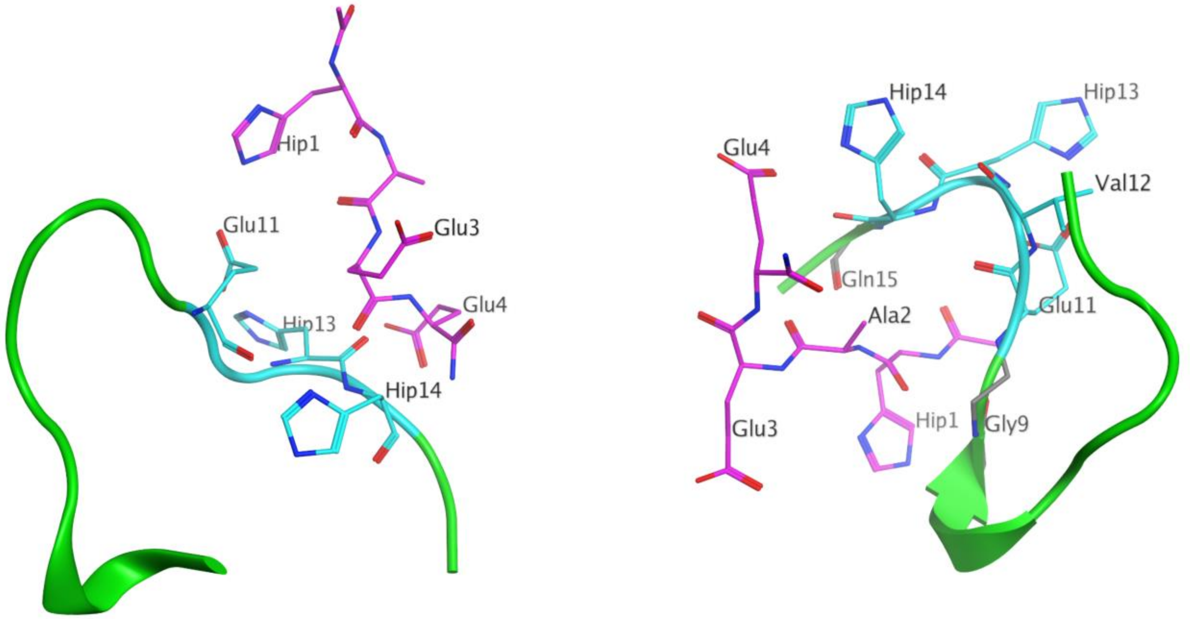

Supplementary Figure 6. **Two Aβ$_{16}$:HAEE complexes with protonated histidines formed transiently during a 50 ns MD simulation.** We previously reported that in the systems of Aβ$_{16}$ (green) with HAEE (magenta) without the zinc ion, the initially formed Aβ$_{16}$:HAEE complexes rapidly break apart. However, in the systems where HAEE drifts away from the Aβ$_{16}$ peptide, we observed that it moved back to the same ^{11}EVHH^{14} interaction site (cyan). This was observed in 9 systems of 12.

© 2022. Mitkevich VA et al. Published online at http://www.aginganddisease.org/EN/10.14336/AD.2022.0827

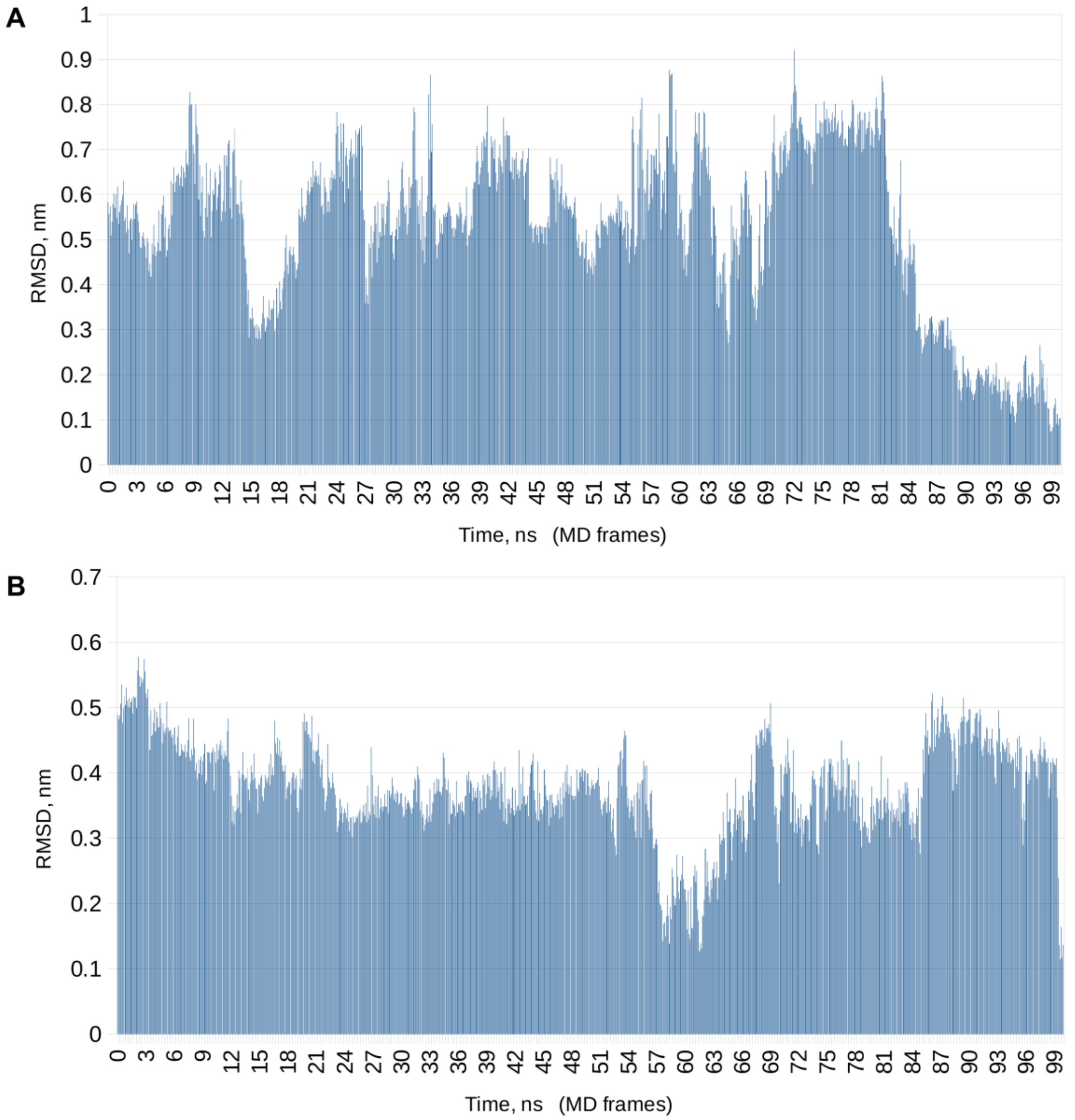

Supplementary Figure 7. RMSD graphs for the backbone of isoD7-Aβ_{16}: HAEE (A) and isoD7-Aβ_{42}: HAEE (B) complexes. Final 100 ns of the 200 ns MD simulation. Calculated as the divergence in the alignment of the structure in every MD frame with the final structure. **(A)** RMSD values were low throughout the simulation and the concluding convergence to the final structure can be clearly seen starting at 84 ns where the RMSD values decreased markedly. **(B)** Conformational changes can be seen in the 55 – 65 ns interval, however the final structure remained stable.

© 2022. Mitkevich VA et al. Published online at http://www.aginganddisease.org/EN/10.14336/AD.2022.0827

SUPPLEMENTARY DATA

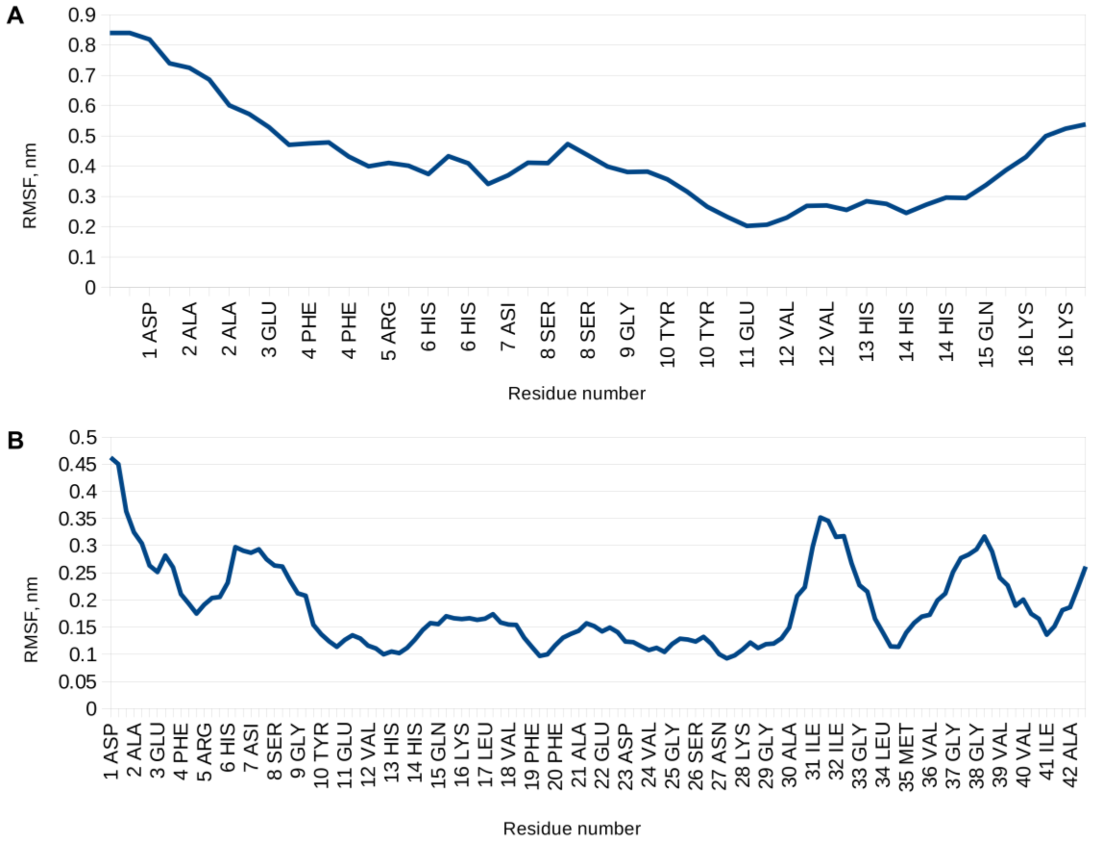

Supplementary Figure 8. RMSF graphs for the backbone of isoD7-Aβ_{16} (A) and isoD7-Aβ_{42} (B) for the final 100 ns of the 200 ns MD simulation trajectory. RMSF indicates the residue flexibility during MD simulation. For both isoD7-Aβ_{16} and isoD7-Aβ_{42} complexes with HAEE coordinated by Zn^{2+}, the region of lower RMSF value corresponds to the ^{11}EVHH^{14} region of the peptides, the most stable and rigid part of the complexes. On the contrary, the N- and C-termini of Aβ peptides were the most flexible.

© 2022. Mitkevich VA et al. Published online at http://www.aginganddisease.org/EN/10.14336/AD.2022.0827

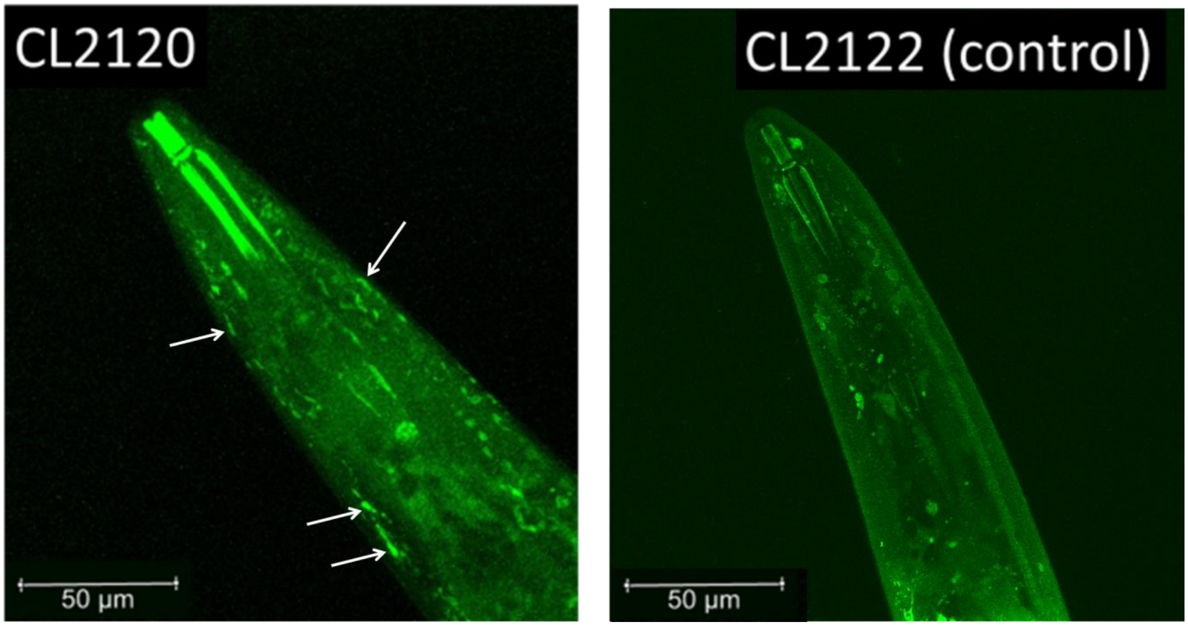

Supplementary Figure 9. Amyloidogenesis in CL2120 transgenic nematodes. Staining was performed with 1 mM X-34 as described in [36]. Amyloid deposits (white arrows) were observed only in the strain with an overexpression of Aβ42 – CL2120. Non-specific diffused green staining was detected in control stain CL2122.

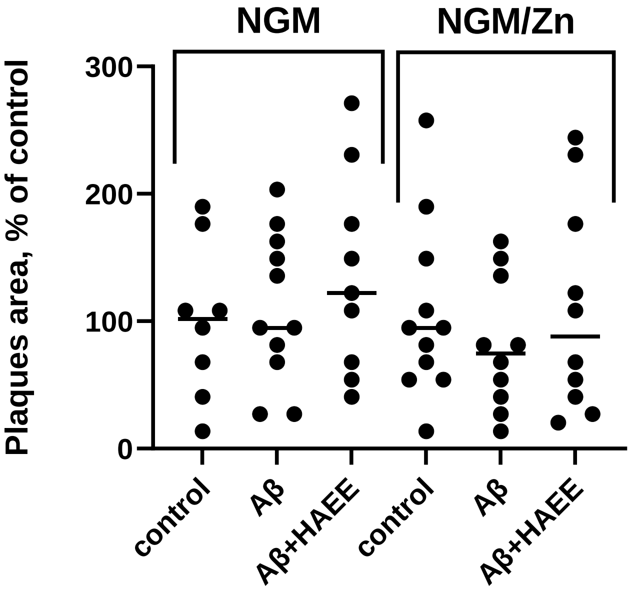

Supplementary Figure 10. Area covered with amyloid aggregates ("plaques") as a percentage of head area, detected by X-34 fluorescence. Worms were treated with 40 μM Aβ42 (Aβ), HAEE, these two peptides combined (Aβ+HAEE) or received no treatment (control). After the incubation with peptides on NGM or NGMZn, the animals were stained with X-34 and the amyloid aggregates were visualized with a confocal microscope. Data shown as individual values with a bar at sample mean. N for control, Aβ, Aβ+HAEE treated worms equals 8, 11, 9 for NGM group and 11, 10, 10 for NGM/Zn group, respectively.

A

© 2022. Mitkevich VA et al. Published online at http://www.aginganddisease.org/EN/10.14336/AD.2022.0827

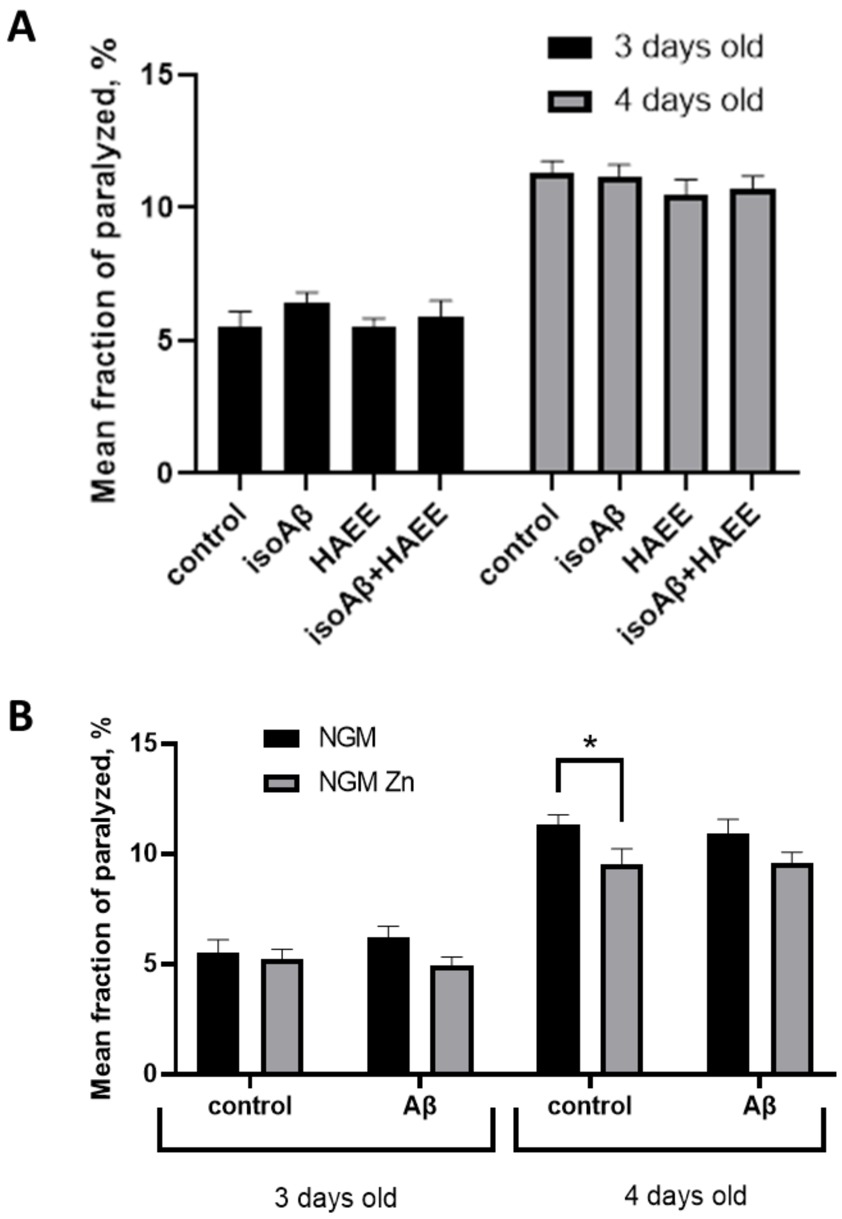

Supplementary Figure 11. (**A**) The effects of isoD7-Aβ_{42} and HAEE on the prevalence of a paralysis phenotype in a *C. elegans* CL2120 of 3 days old (black) and 4 days old (gray). Treatment: 0.2 ml M9 buffer +/- isoD7-Aβ_{42} (40 µM) +/- HAEE (4 mM). Strains were grown on an NGM medium. Data shown as the mean of 3 independent experiments ± SD. (**B**) The effect of Aβ_{42} on the prevalence of a paralysis phenotype in *C. elegans* CL2120 of 3 days old and 4 days old, grown on a NGM (black) or NGMZn (gray) medium. Treatment: 0.2 ml M9 buffer +/- $ZnSO_4$ +/- Aβ42 (40 µM). Data shown as the mean of 3 independent experiments ± SD. Brackets represent statistically significant comparisons according to ANOVA with post-hoc Tukey test. * - $p<0.05$.

© 2022. Mitkevich VA et al. Published online at http://www.aginganddisease.org/EN/10.14336/AD.2022.0827

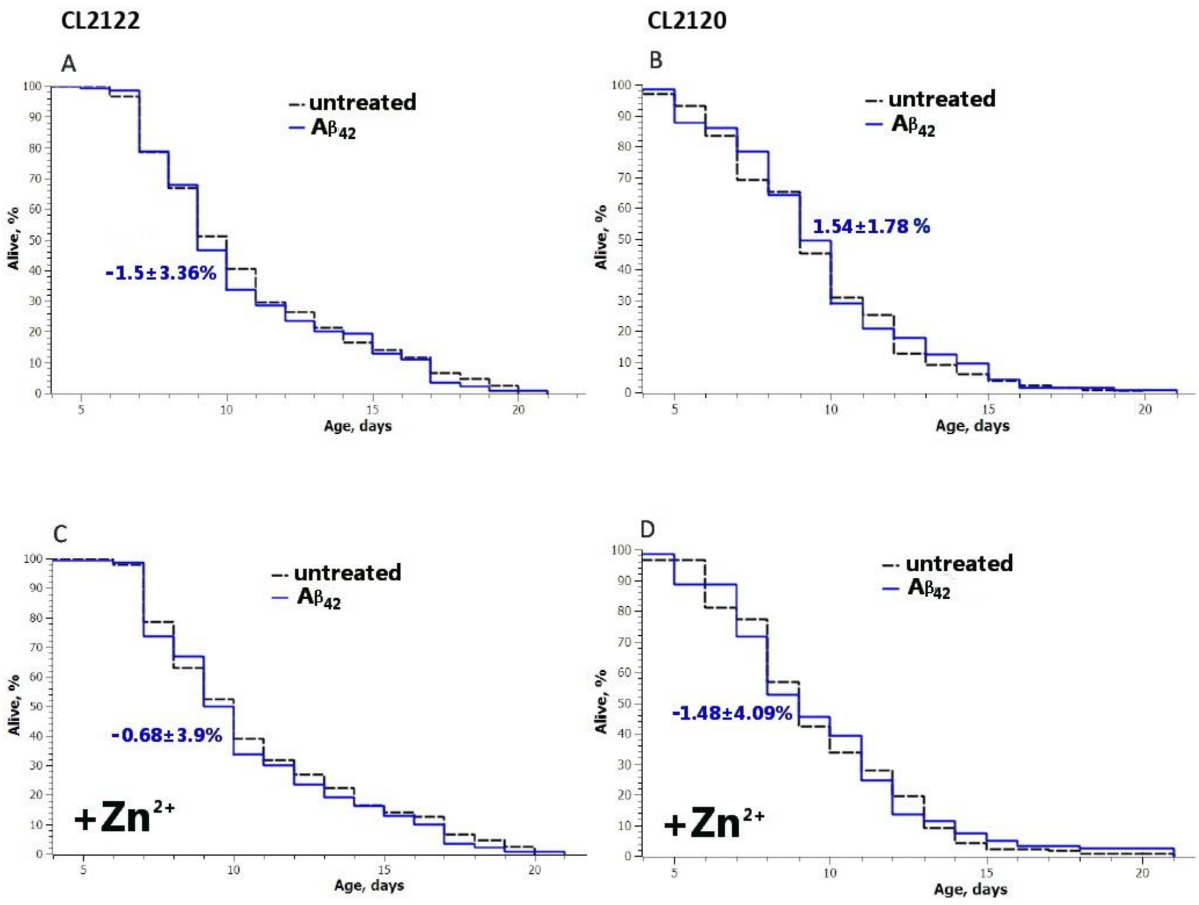

Supplementary Figure 12. The effect of exogenous Aβ$_{42}$ on the lifespan of model animals. The graphs plotting and processing by the method of sigmoidal approximation of the experimental data, were carried out using the SciDAVis software package for statistical analysis. All lifespan plots represent the composites of 3 independent experiments tabulated in Supplementary Table 3. Mean lifespans were compared using the Student t test, applying one-tailed distribution and two-sample equal variance. The average change ± SD of the lifespan (percent) is shown relative to the control on each graph. **A** and **B,** Exogenous Aβ$_{42}$ peptide does not affect the lifespan of control animals CL2122 (**A**) and nematodes with endogenous Aβ42 CL2120 (**B**). Nematodes were grown on NGM until stage L4, treated with Aβ$_{42}$ (40 μM), then worms were grown on NGM. **C** and **D**, Simultaneous treatment with the Aβ$_{42}$ peptide and zinc ions does not affect the lifespan of model animals: CL2122 (**C**) and CL2120 (**D**). After the treatment with Aβ$_{42}$ (40 μM) + $ZnSO_4$ (20 μM) nematodes were grown on NGMZn.

© 2022. Mitkevich VA et al. Published online at http://www.aginganddisease.org/EN/10.14336/AD.2022.0827

SUPPLEMENTARY DATA

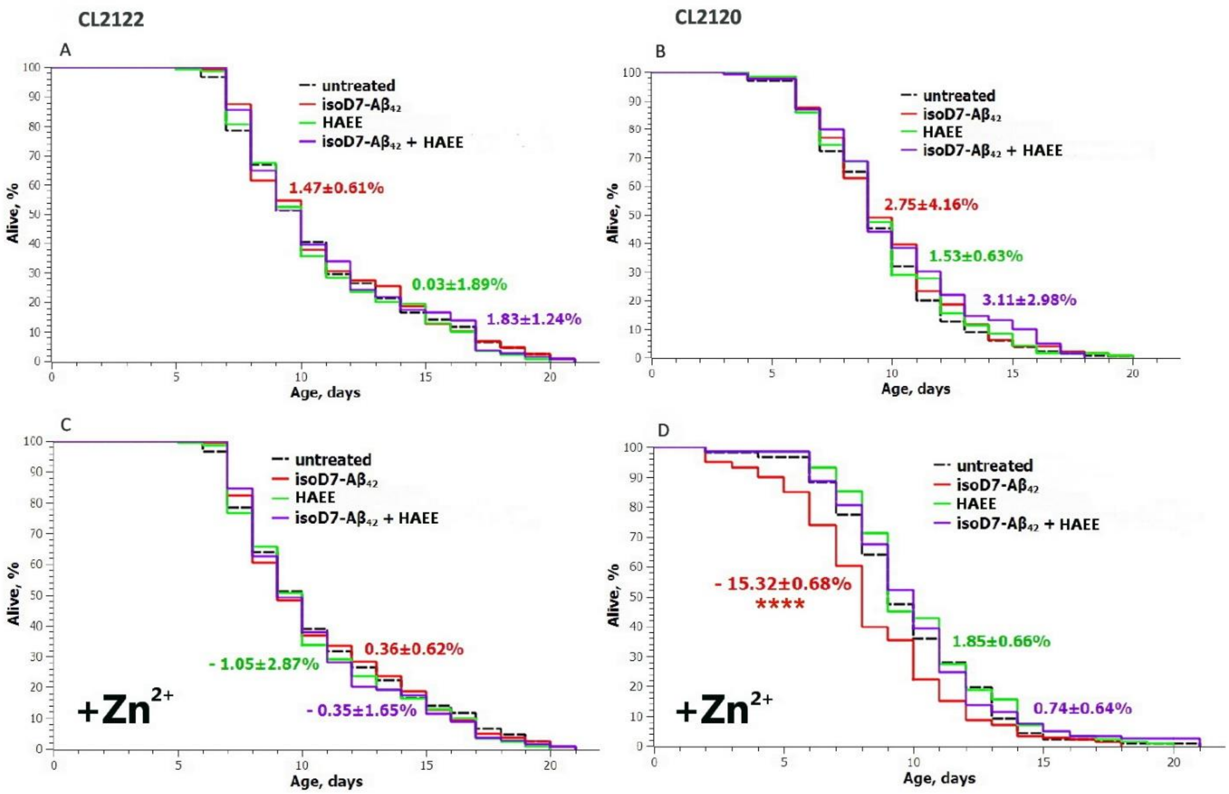

Supplementary Figure 13. The effect of exogenous isoD7-Aβ42, Zn^{2+} and HAEE on lifespan of model animals. The graph plotting and processing by the method of sigmoidal approximation of the experimental data, were carried out using the SciDAVis software package for statistical analysis. All lifespan plots represent the composites of 3 independent experiments tabulated in Supplementary Table 4. Mean lifespans were compared using the Student t test, applying one-tailed distribution and two-sample equal variance. Mean percentage change ± SD of the lifespan after treatment relative to untreated control is indicated in each graph in the same color as the curve. **A** and **B,** The isoD7-Aβ42 peptide and HAEE tetrapetide do not affect the lifespan of control animals CL2122 (**A**) and nematodes with endogenous Aβ42 CL2120 (**B**). Nematodes were grown on NGM until stage L4. Treatment: isoD7-Aβ42 (40 µM) +/- HAEE (4 mM). Then nematodes were grown on NGM. **C** and **D,** Simultaneous treatment with the isoD7-Aβ42 peptide and zinc ions does not affect the lifespan of control animals CL2122 (**C**), but reduces the lifespan of the endogenous Aβ42 nematodes CL2120 (**D**). The HAEE tetrapeptide removes the negative effect of the simultaneous addition of the isoD7-Aβ42 peptide and zinc ions on the lifespan of CL2120 nematodes (**D**). Nematodes were grown on NGM until stage L4, treated with $ZnSO_4$ (20 µM) +/- isoD7-Aβ42 (40 µM) +/- HAEE (4 mM), the lifespan was determined on NGMZn. **** - $p<0.0001$

Supplementary Table 1. Affinity and kinetic parameters of interactions between immobilized Aβ42 or isoD7-Aβ42 and HAEE in the presence of 100 µM $ZnCl_2$ at pH 6.8, obtained by SPR[a].

Complex	k_{on}, $M^{-1}s^{-1}$ [b]	k_{off}, s^{-1} [b]	K_D, M [c]	χ^2 [d]
Aβ42/HAEE	$(1.42\pm0.06) \times 10^3$	$(5.75\pm0.06) \times 10^{-3}$	$(4.1\pm0.3) \times 10^{-6}$	2.43
isoD7-Aβ42/HAEE	$(6.7\pm0.2) \times 10^2$	$(6.94\pm0.07) \times 10^{-3}$	$(1.04\pm0.04) \times 10^{-5}$	3.01

[a] All the parameters have been calculated using sets of sensorgrams obtained during serial injections of the analytes with different concentrations. Data represent mean ± SD of three independent experiments.
[b] Association (k_{on}) and dissociation (k_{off}) rate constants were calculated using the Langmuir binding model (1:1 complex formation) with fitting of model and experimental curves.
[c] Equilibrium dissociation (K_D) constants for the complexes were calculated as the ratio: $K_D = k_{off}/k_{on}$.
[d] Chi-square values were calculated with **BIAevaluation v.4.1 software** using the obtained sets of sensorgrams**.**

Supplementary Table 2. Paralysis of *Caenorhabditis elegans* CL2120. Treatment of Aβ42, isoD7- Aβ42, and HAEE.

© 2022. Mitkevich VA et al. Published online at http://www.aginganddisease.org/EN/10.14336/AD.2022.0827

SUPPLEMENTARY DATA

Rep-eats	Age, days	Media/treatment	Number of animals that are included in the experiment/total	Fraction of paralyzed, %	Mean fraction of paralyzed, %±SD	P-value
1	A3	NGM	110/115	5.3	5.5±0.59	
2			108/110	6.2		
3			110/112	5.1		
1		NGM +Aβ42	115/117	5.6	6.2±0.51	0.11590
2			106/110	6.3		
3			118/120	6.6		
1		NGM +isoD7-Aβ42	112/115	6.3	6.4±0.42	0.04798
2			117/120	6.9		
3			108/110	6.1		
1		NGM +HAEE	105/110	5.5	5.5±0.35	0.5
2			108/110	5.2		
3			106/110	5.9		
1		NGM + isoD7- Aβ42 + HAEE	127/130	6.5	5.9±0.6	0.24552
2			110/115	5.3		
3			115/117	5.9		
1		NGMZn	110/115	5.6	5.2±0.46	
2			108/110	5.3		
3			107/110	4.7		
1		NGMZn + Aβ42	112/115	5.3	4.9±0.4	0.19910
2			106/115	4.8		
3			114/115	4.5		
1		NGMZn + isoD7- Aβ42	111/115	10.8	11.3±0.4	0.00003
2			113/115	11.5		
3			104/110	11.5		
1		NGM Zn+ isoD7- Aβ42 + HAEE	122/125	5.7	5.4±0.29	0.31113
2			115/118	5.2		
3			116/120	5.2		
1		NGMZn+ isoD7- Aβ42 + HAEE	106/110	8.2	7.6±0.49	0.00166
2			108/110	7.3		
3			120/125	7.4		
1	A4	NGM	110/115	11.3	11.3±0.45	
2			108/110	11.7		
3			110/112	10.8		
1		NGM +Aβ42	115/117	10.8	10.9±0.66	0.23478
2			106/110	11.6		
3			118/120	10.3		
1		NGM +isoD7- Aβ42	112/115	11.7	11.2±0.42	0.46479
2			117/120	10.9		
3			108/110	11.1		
1		NGM +HAEE	105/110	9.9	10.5±0.55	0.07446
2			108/110	10.9		
3			106/110	10.8		
1		NGM + isoD7- Aβ42 + HAEE	127/130	10.1	10.7±0.49	0.09746
2			110/115	11		

© 2022. Mitkevich VA et al. Published online at http://www.aginganddisease.org/EN/10.14336/AD.2022.0827

3			115/117	10.9		
1		NGMZn	110/115	10.1	9.5±0.72	
2			108/110	9.7		
3			107/110	8.7		
1		NGMZn + Aβ42	112/115	9.4	9.6±0.47	0.44996
2			106/115	10.1		
3			114/115	9.2		
1		NGMZn + isoD7-Aβ42	111/115	22.5	21.8±0.7	0.00001
2			113/115	21.8		
3			104/110	21.1		
1		NGMZn + HAEE-	122/125	10.2	9.5±0.65	0.47772
2			115/118	9.5		
3			116/120	8.9		
1		NGMZn+ isoD7- Aβ42 + HAEE	106/110	11.8	11.2±0.53	0.01508
2			108/110	10.8		
3			120/125	11		

The mean fraction of paralyzed animals of 3-days old (A3) or 4 days old (A4). Independent experimental and control analyses, which were performed side-by-side, are indicated by the same number (1, 2 or 3) in the first column. P-values of pairwise comparisons with control groups (NGM or NGMZn in absence of β-amyloid peptides) are shown.

© 2022. Mitkevich VA et al. Published online at http://www.aginganddisease.org/EN/10.14336/AD.2022.0827

SUPPLEMENTARY DATA

Supplementary Table 3. Experiments performed to study the effect of Aβ_{42} peptide on the lifespan of nematodes.

Rep-eats	*C. elegans*	Media/ treatment	Number of animals that died/total	50% survival, days	Mean survival, days±SD	P-value, 1 2	Increase/ decrease, %	Mean increase/ decrease, %±SD
1	CL2120	NGM	120/135	8.6	8.67±0.21			
2			125/130	8.9				
3			128/140	8.5				
1		NGM + Aβ_{42}	135/155	8.9	8.8±0.26	0.26521	3.49	1.54±1.78
2			123/140	9.0			1.12	
3			122/145	8.5			0	
1		NGMZn	135/155	8.3	8.7±0.4			
2			138/160	8.7				
3			125/140	9.1				
1		NGMZn + Aβ_{42}	131/145	8.5	8.57±0.4	0.35271	2.41	-1.48±4.09
2			123/145	8.2			-5.75	
3			139/155	9.0			-1.10	
1	CL2122	NGM	135/150	9.2	9.07±0.12			
2			123/145	9.0				
3			139/150	9.0				
1		NGM + Aβ_{42}	140/155	9.4	8.93±0.42	0.31065	2.17	-1.50±3.36
2			138/150	8.6			-4.44	
3			136/150	8.8			-2.22	
1		NGMZn	138/155	8.8	9.03±0.21			
2			123/140	9.2				
3			134/145	9.1				
1		NGMZn + Aβ_{42}	141/150	9.1	8.97±0.15	0.33893	3.41	-0.68±3.9
2			132/145	8.8			-4.35	
3			135/150	9.0			-1.10	

Each data set (repeat) is fitted to a Boltzmann sigmoid curve and the mean survival time calculated. The % change in lifespan is with respect to the control in the same repeat. The independent experimental and control analyses, which were performed side-by-side, are indicated by the same number (1, 2 or 3) in the first column. Increase (+) or decrease (-) in the lifespan is indicated. P-values have been calculated with respect to the control animals in the same experiment using Student's t-test (one-tailed distribution and two-sample equal variance).

SUPPLEMENTARY DATA

Supplementary Table 4. Experiments performed to study the effect of isoD7-$A\beta_{42}$ peptide and the HAEE tetrapetide on the lifespan of nematodes.

Rep-eats	*C. elegans*	Media/treatment	Number of animals that died /total	50% survival, days	Mean survival, days±SD	P-value, 1 2	Increase/ decrease, %	Mean increase/ decrease, %±SD
1	CL2120	NGM	121/130	8.4	8.7±0.26			
2			134/145	8.8				
3			131/145	8.9				
1		NGM + isoD7-$A\beta_{42}$	121/135	9	8.93±0.21	0.14809	7.14	2.75±4.16
2			130/140	8.7			-1.14	
3			138/148	9.1			2.25	
1		NGM + HAEE	141/150	8.5	8.83±0.31	0.29914	1.19	1.53±0.63
2			139/150	8.9			1.14	
3			136/145	9.1			2.25	
1		NGM + isoD7-$A\beta_{42}$ + HAEE	126/135	8.9	8.97±0.21	0.12099	5.95	3.11±2.98
2			142/150	8.8			0	
3			139/150	9.2			3.37	
1		NGMZn	137/150	9.2	9±0.16			
2			122/135	8.8				
3			139/145	9.1				
4			120/135	9.0				
5			131/145	8.9				
1		NGMZn + isoD7-$A\beta_{42}$	128/135	7.7	7.62±0.08	0.0000001	-16.3	-15.32 ±0.68
2			133/140	7.5			-14.77	
3			121/135	7.7			-15.38	
4			130/144	7.6			-15.56	
5			123/135	7.6			-14.61	
1		NGMZn + HAEE	141/150	9.3	9.2±0.7	0.07227	1.09	1.85 ±0.66
2			128/135	9			2.27	
3			133/145	9.3			2.20	
1		NGMZn + isoD7-$A\beta_{42}$ + HAEE	122/135	9.3	9.1±0.2	0.22965	1.09	0.74 ±0.64
2			133/145	8.9			1.14	
3			135/145	9.1			0	
1	CL2122	NGM	129/140	8.7	9.07±0.32			
2			137/150	9.2				
3			139/150	9.3				
1		NGM + isoD7-$A\beta_{42}$	124/135	8.8	9.2±0.35	0.32533	1.15	1.47±0.61
2			141/150	9.4			2.17	
3			142/150	9.4			1.08	
1		NGM + HAEE	131/140	8.8	9.07±0.25	0.5	1.15	0.03±1.89
2			138/150	9.3			1.09	
3			142/150	9.1			-2.15	
1		NGM + isoD7-$A\beta_{42}$ + HAEE	129/140	8.8	9.23±0.38	0.29613	1.15	1.83±1.24
2			142/150	9.5			3.26	
3			141/150	9.4			1.08	
1		NGMZn	122/135	8.7	9.03±0.31			

© 2022. Mitkevich VA et al. Published online at http://www.aginganddisease.org/EN/10.14336/AD.2022.0827

SUPPLEMENTARY DATA

2			135/145	9.1				
3			144/150	9.3				
1		NGMZn + isoD7-Aβ42	142/150	8.7	9.07±0.35	0.45364	0	0.36 ±0.62
2			145/150	9.1			0	
3			142/150	9.4			1.08	
1		NGMZn + HAEE	141/150	8.8	8.93±0.15	0.31938	1.15	-1.05 ±2.87
2			142/150	9.1			0	
3			134/145	8.9			-4.3	
1		NGMZn + isoD7-Aβ42+ HAEE	143/150	8.7	9±0.26	0.44666	0	-0.35 ±1.65
2			131/140	9.2			1.10	
3			141/150	9.1			-2.15	

Each data set (repeat) was fitted to a Boltzmann sigmoid curve and the mean survival time calculated. The % change in lifespan is with respect to the control in the same repeat. Independent experimental and control analyses, which were performed side-by-side, are indicated by the same number (1, 2, 3, 4 or 5) in the first column. Increase (+) or decrease (-) in lifespan is indicated. P-values have been calculated with respect to the control animals in the same experiment using Student's t-test (one-tailed distribution and two-sample equal variance).

© 2022. Mitkevich VA et al. Published online at http://www.aginganddisease.org/EN/10.14336/AD.2022.0827
